# Supplementary material for: Pathological complete response of adding targeted therapy to neoadjuvant chemotherapy for inflammatory breast cancer: A systematic review
Source: PLoS One. 2021 Apr 16;16(4):e0250057. doi: 10.1371/journal.pone.0250057 (PMC8051801; doi:10.1371/journal.pone.0250057)
Supplement: S1 Table — (DOCX) [file pone.0250057.s001.docx]

**S1 Table.** Search strategy Ovid MEDLINE (July 15, 2020)

| # | Searches | Results |
| --- | --- | --- |
| 1 | *Inflammatory Breast Neoplasms/ | 407 |
| 2 | (inflammat* and breast).ti,kw. | 2021 |
| 3 | (inflammat* and breast).ab. /freq=2 | 4356 |
| 4 | 1 or 2 or 3 | 5535 |
| 5 | limit 4 to (yr=”1998 – 2020” and clinical trial, all) | 275 |
