# Supplementary material for: Pathological complete response of adding targeted therapy to neoadjuvant chemotherapy for inflammatory breast cancer: A systematic review
Source: PLoS One. 2021 Apr 16;16(4):e0250057. doi: 10.1371/journal.pone.0250057 (PMC8051801; doi:10.1371/journal.pone.0250057)
Supplement: S2 Table — (DOCX) [file pone.0250057.s002.docx]

**S2 Table.** Risk of bias by ROBIN-I

| **First author/reference** | **Baseline confounding** | **Selection bias** | **Classification of intervention** | **Deviations from intervention** | **Missing data** | **Measurement of outcomes** | **Selection of reported results** | **Overall risk of bias** | |
| --- | --- | --- | --- | --- | --- | --- | --- | --- | --- |
| Pizzuti [37] | Low | Low | Low | Low | Low | Low | Low | Low | |
| Torrisi [34] | Low | Low | Low | Low | Low | Low | Low | Low | |
| Palazzo [35] | Low | Moderate | Low | Low | Low | Low | Low | Moderate | |
| Pierga [36] | Low | Low | Low | Low | Low | Low | Low | Low | |
| Nahleh [31] | Moderate | Low | Low | Low | Low | Low | Low | Moderate | |
| Bertucci [33] | Low | Low | Low | Moderate | Low | Low | Low | Moderate | |
| Matsuda [32] | Low | Low | Low | Low | Low | Low | Low | Low | |
| Boussen [39] | Moderate | Moderate | Low | Moderate | Low | Low | Low | Moderate | |
| Andreopoulou [38] | Serious | Low | Low | Low | Low | Low | Low | Serious | |
| Cristofanilli [45] | Moderate | Serious | Low | Low | Low | Low | Low | Serious | |
| de Matteis [46] | Moderate | Low | Low | Low | Low | Low | Low | Moderate | |
| Ditsch [41] | Low | Low | Low | Low | No information | Low | Low | Low | |
| Baldini [44] | Low | Low | Moderate | Low | No information | Low | Low | Moderate | |
| Veyret [42] | Low | Low | Low | Low | Low | Low | Low | Low | |
| Kummel [43] | Low | Low | Low | Low | Low | Low | Low | Low | |
| Costa [40] | Low | Serious | Low | Low | Low | Low | Low | Serious | |
| Ellis [20] | Low | Low | Low | Low | Low | Low | Low | Low | |
| Nahleh [31] | Mod | Low | Low | Low | Low | Low | Low | Moderate | |
| Schwartzberg [27] | Mod | Low | Low | Low | Low | Low | Low | Moderate | |
| Sportes [28] | Low | Low | Low | Low | Low | Low | Low | Low | |
| P Viens [30] | Low | Low | Low | Low | Low | Low | Low | Low | |
| PEGASE 02 trial [29] | Low | Low | Low | Low | Low | Low | Low | Low | |
| Dazzi [25] | Moderate | Low | Serious | Low | Low | Low | Low | Serious | |
| Goncalves [26] | Low | Low | Low | Low | Low | Low | Low | Low |  |
