# Supplementary material for: Pathological complete response of adding targeted therapy to neoadjuvant chemotherapy for inflammatory breast cancer: A systematic review
Source: PLoS One. 2021 Apr 16;16(4):e0250057. doi: 10.1371/journal.pone.0250057 (PMC8051801; doi:10.1371/journal.pone.0250057)
Supplement: S3 Table — (DOCX) [file pone.0250057.s003.docx]

**S3 Table.** Characteristics of the included studies and baseline characteristics of the patients in high-dose chemotherapy studies

| **First author/**  **reference** | **Phase** | **Inclusion criteria** | **Total**  **(No.)** | **HER2+**  **(No.)** | **ER+**  **(No.)** | **PR+**  **(No.)** | **HR+**  **(No.)** | **IBC**  **(No.)** | **Median age (range), y** | **Years of accrual** | **Risk of bias** | |
| --- | --- | --- | --- | --- | --- | --- | --- | --- | --- | --- | --- | --- |
| **High-dose chemotherapy** | | | | | | | | | | | |  |
| Schwartzberg [27] | 3 | III | 41 | NR | NR | NR | NR | 41 | 47 | 1992-1997 | Moderate | |
|  |  |  |  |  |  |  |  |  | (26-63) |  |  |  |
| Sportes [28] | 2 | III | 21 | 13 | NR | NR | 11 | 21 | 50 | 1996-2008 | Low | |
|  |  |  |  |  |  |  |  |  | (35-67) |  |  |  |
| Viens [30] | 2 | III | 17 | 2 | 8 | 7 | 9 | 17 | 46 | NR | Low | |
|  |  |  |  |  |  |  |  |  | (24-54) |  |  |  |
| Viens | 2 | III | 95 | NR | NR | NR | 33 | 95 | 46 | 1994-1996 | Low | |
| PEGASE 02 trial [29] |  |  |  |  |  |  |  |  | (26-59) |  |  |  |
| Dazzi [25] | 2 | III | 21 | NR | NR | NR | NR | 21 | 46 | 1994-1998 | Serious | |
|  |  |  |  |  |  |  |  |  | (29-56) |  |  |  |
| Goncalves [26] | 3 | III | 174 | 33 | 72 | NR | 78 | 174 | 45 | 2001-2005 | Low | |
|  |  |  |  |  |  |  |  |  | (25-56) |  |  |  |
