# Supplementary material for: Pathological complete response of adding targeted therapy to neoadjuvant chemotherapy for inflammatory breast cancer: A systematic review
Source: PLoS One. 2021 Apr 16;16(4):e0250057. doi: 10.1371/journal.pone.0250057 (PMC8051801; doi:10.1371/journal.pone.0250057)
Supplement: S4 Table — (DOCX) [file pone.0250057.s004.docx]

**S4 Table.** pCR rates in each study of high-dose chemotherapy studies.

| **First author/reference** | **IBC patients**  **(No.)** | **Non-IBC patients**  **(No.)** | **Regimen** | **pCR rate, %** | | |
| --- | --- | --- | --- | --- | --- | --- |
|  |  |  |  | **IBC** | **Non-IBC** | **Overall** |
| **High-dose chemotherapy** | | | | | | |
| Schwartzberg [27] | 41 | 0 | Neoadj. HD: cyclophosphamide/thiotepa/carboplatin → HSCS → surgery | 17† | NR | NR |
|  |  |  |  |  |  |  |
| Sportes [28] | 21 | 0 | Neoadj. paclitaxel/cyclophosphamide → AC → HD: melphalan/etoposide → HSCS → surgery | 9 | NR | NR |
| Viens [30] | 17 | 0 | Neoadj. FAC → HD: mitoxantrone/melphalan/ cyclophosphamide → HSCS → surgery | 39† | NR | NR |
|  |  |  |  |  |  |  |
| Viens PEGASE 02 [29] | 95 | 0 | Neoadj. HD: FAC → HSCS → surgery | 32† | NR | NR |
| Dazzi [25] | 21 | 0 | Neoadj. epirubicin → HD: mitoxantrone/thiotepa /cyclophosphamide → HSCS → surgery | 21 | NR | NR |
| Goncalves [26] | 174 | 0 | Neoadj. HD: epirubicin/cyclophosphamide → HSCS → surgery | 20.10 | NR | NR |
|  |  |  | Arm 1: no adjuvant chemotherapy |  |  |  |
|  |  |  | Arm 2: adjuvant docetaxel/5-FU |  |  |  |

†pCR was defined as absence of invasive cancer cells in breast specimen.
