# Supplementary figures and images for: Pathological complete response of adding targeted therapy to neoadjuvant chemotherapy for inflammatory breast cancer: A systematic review
Source: PLoS One. 2021 Apr 16;16(4):e0250057. doi: 10.1371/journal.pone.0250057 (PMC8051801; doi:10.1371/journal.pone.0250057)

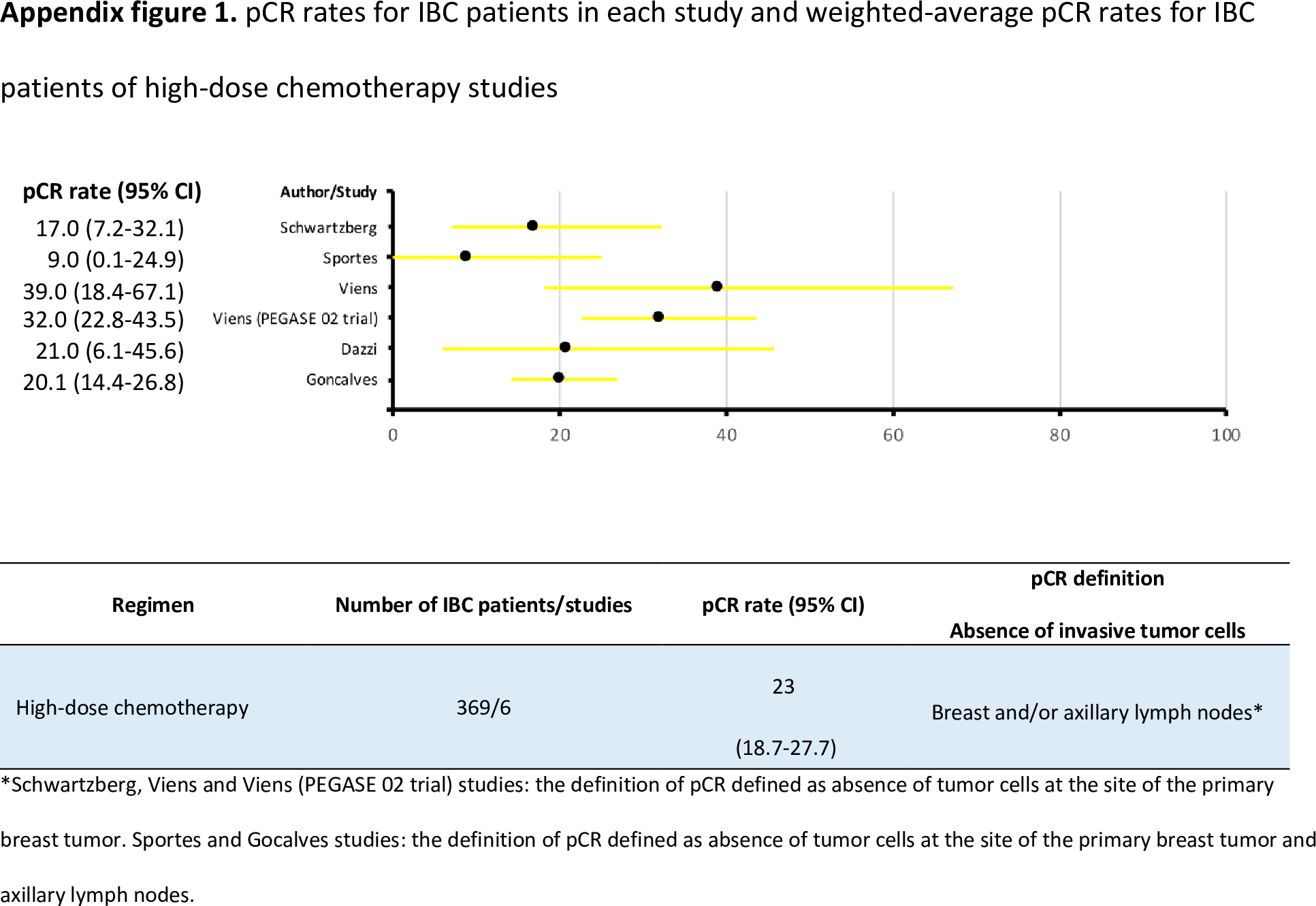

Supplement: S1 Fig — (TIF) [file pone.0250057.s006.tif]
