# Supplementary material for: Pathological complete response of adding targeted therapy to neoadjuvant chemotherapy for inflammatory breast cancer: A systematic review
Source: PLoS One. 2021 Apr 16;16(4):e0250057. doi: 10.1371/journal.pone.0250057 (PMC8051801; doi:10.1371/journal.pone.0250057)
Supplement: S1 Appendix — (DOCX) [file pone.0250057.s007.docx]

**S1 Appendix.**

**High-Dose Chemotherapy**

Our review included 6 studies [25-30] of high-dose chemotherapy that included 369 IBC patients. Because the studies were conducted a long time ago, breast cancer subtypes (e.g., HER2 status) were not available. Moreover, 3 studies [27, 29, 30] defined pCR as lack of viable tumor cells in the breast specimen, which differs from the current definition of lack of invasive tumor cells in the breast and axillary lymph node specimens. The pCR rates ranged from 9.0% to 39.0%. The lowest pCR rate was observed in a study by Sportes et al. [28], in which patients were treated with paclitaxel plus cyclophosphamide followed by doxorubicin and cyclophosphamide and then high-dose chemotherapy with melphalan and etoposide with HSCS. The highest pCR rate was observed in a study by Veins et al. [30], in which patients were treated with 5-fluorouracil, doxorubicin, and cyclophosphamide for 4 cycles followed by high-dose chemotherapy with mitoxantrone, melphalan, and cyclophosphamide with HSCS, and then surgery.
