# Supplementary material for: Pathological complete response of adding targeted therapy to neoadjuvant chemotherapy for inflammatory breast cancer: A systematic review
Source: PLoS One. 2021 Apr 16;16(4):e0250057. doi: 10.1371/journal.pone.0250057 (PMC8051801; doi:10.1371/journal.pone.0250057)
Supplement: Attachment — Submitted filename: Reviewer comments.docx [file pone.0250057.s008.docx]

**Reviewer comments**

This paper reports on the pathological complete response rates with addition of targeted therapy to neoadjuvant chemotherapy for inflammatory breast cancer. It is an extensive review on a rare subtype of breast cancer. However, I have following concerns -

**Methods**

Outcome measure –

The main outcome of study is pathological complete response (path CR). However, the authors have considered two definitions of path CR - “the proportion of patients with absence of invasive cancer cells from breast or breast and axillary lymph nodes after neoadjuvant systemic treatment.’’

The current standard definition of path CR is absence of cancer cells from both breast and axillary Lymph nodes. Why have the authors taken the old definition of path CR, which is likely to overestimate the path CR rates?

**Data Synthesis and analysis**

High dose chemotherapy and hematopoietic stem cell support is not the current standard of care for breast cancer. The definition of path CR in high dose chemotherapy group is not as per the current standard, at best these studies overestimates the benefit of chemotherapy and the path CR rates. Moreover no targeted therapies were used in this group of trials. Since the aim of the present systemic review is to look at the improvement in path CR rates with addition of targeted therapies in inflammatory breast cancer patients (IBC), it would be better to drop the high dose chemotherapy group from the analysis so as to have a more homogenous comparator. Previous systemic review by Kim T et al (Clin Breast Cancer. 2006) has already addressed the path CR rates and issues with high dose chemotherapy in inflammatory breast cancer.

**Study characteristics**

Patients with locally recurrent IBC were included in the analysis. Since locally recurrent breast cancer have different disease biology and may not have the same response rates as in treatment naïve patients, their inclusion might have underestimated the path CR rates in both comparator and intervention arm. Request comment from authors on the same.

**Results:**

Pathological CR rates depend upon the breast cancer subtypes (Her 2 positive, TNBC and hormone receptor positive) and on the type of systemic therapies given. In this review all subtypes were clubbed together particularly in the comparator arm (chemotherapy alone group), which could have underestimated the path CR rates in this group. It would be better if the path CR rates can be given according to the breast cancer subtypes.

Similarly the weighted averages pCR rates are reported for all targeted therapies as a single group, where in panitumumab and tipifarnib are for Her2 negative breast cancer and trastuzumab is for Her2 positive breast cancer. It would be better to report the path CR rates with targeted therapies for each breast cancer subtype separately rather than as a single group.

**Discussion:**

Para 1 - “There was a difference of 18.6 percentage points between the weighted-average pCR for chemotherapy alone and the weighted-average pCR for targeted therapy plus chemotherapy”, this is probably an overstatement by authors since the weighted averages pCR rates are not as per the breast cancer subtypes. Both the targeted therapy and chemotherapy alone groups are heterogeneous and it is not correct to compare the biologically different subtypes directly. Would request a comment on this.

Para 4, 5 - Given the lack of benefit of bevacizumab in any breast cancer subtype, putting much emphasis on the role of bevacizumab in discussion (2 paragraphs have been written on this) seems excessive. Simply stating that addition of bevacizumab did not improve path CR in any breast cancer subtype would suffice.

Para 6 – weighted average path CR rates were calculated for non IBC patients as well. The methods section did not mention about this for non -IBC patients, moreover the primary aim of review is to analyze the path CR rates in IBC patients. Also, the search criteria included inflammatory breast cancer only; hence the calculation of weighted average path CR in non IBC patients from highly select group of trials may not be the ideal way and will not represent the true path CR rates in non IBC patients.

Para 9 – the schedule of chemotherapy and dose intensity of chemotherapy affect the path CR rates, which has not been analyzed in present study. Notably, 2 trials looked at efficacy of dose dense chemotherapy versus standard dose in IBC; both these trials have been included (ref 20, 35) but not analyzed from this point of view.
